# Single-Digit Nanometer Electron-Beam Lithography with an Aberration-Corrected Scanning Transmission Electron Microscope

**DOI:** 10.3791/58272

**Published:** 2018-09-14

**Authors:** Fernando E. Camino, Vitor R. Manfrinato, Aaron Stein, Lihua Zhang, Ming Lu, Eric A. Stach, Charles T. Black

**Affiliations:** ^1^Center for Functional Nanomaterials, Brookhaven National Laboratory

**Keywords:** Engineering, Issue 139, Nanofabrication, electron-beam lithography, aberration correction, electron microscopy, nanomaterials, pattern transfer, e-beam resist, poly (methyl methacrylate), hydrogen silsesquioxane

## Abstract

We demonstrate extension of electron-beam lithography using conventional resists and pattern transfer processes to single-digit nanometer dimensions by employing an aberration-corrected scanning transmission electron microscope as the exposure tool. Here, we present results of single-digit nanometer patterning of two widely used electron-beam resists: poly (methyl methacrylate) and hydrogen silsesquioxane. The method achieves sub-5 nanometer features in poly (methyl methacrylate) and sub-10 nanometer resolution in hydrogen silsesquioxane. High-fidelity transfer of these patterns into target materials of choice can be performed using metal lift-off, plasma etch, and resist infiltration with organometallics.

**Figure:**
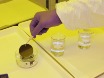


## Introduction

The protocol presented in this manuscript provides guidance for defining patterns with single-digit nanometer resolution in poly (methyl methacrylate) (PMMA) and hydrogen silsesquioxane (HSQ), which are two common electron-beam resists used in high-resolution patterning by electron-beam lithography. We achieve these results using an aberration-corrected scanning transmission electron microscope (STEM) as the exposure tool, outfitted with a pattern generator for controlling the electron beam. After resist exposure, the nanoscale patterns can be transferred to a variety of target materials^1^, thus enabling fabrication of novel devices at single-digit nanometer resolution.

Previous studies have shown that electron-beam lithography (EBL) is capable of defining patterns in resist materials with dimensions in the sub-10 nm scale^2^,^3^,^4^,^5^,^6^. However, for dimensions around 4 nm, these demonstrations have required non-standard procedures such as use of assist structures^7^ or long-exposure times for self-developing resists^8^. Other nanopatterning techniques, such as electron-beam induced deposition^9^ or scanning probe lithography^10^,^11^, have proven capable of achieving sub-4 nm resolution, although these require significantly longer exposure times compared to EBL.

Modern dedicated EBL systems produce electron beams with spot sizes in the few nanometer length scale (2-10 nm), which makes defining patterns with sub-10 nm resolution very difficult. In contrast, our protocol implements EBL using an aberration-corrected STEM, which is a highly optimized instrument for material characterization at angstrom length scales. This difference allows routine patterning of record-breaking lithographic features with single nanometer resolution^1^. While state-of-the-art, commercial aberration-corrected STEM systems cost in the range of millions of dollars, they are available for use in several national user facilities, and some are accessible without cost.

## Protocol

### 1. Sample Preparation for Resist Coating

Note: In this work, patterns with single-digit nanometer resolution are defined in PMMA (positive- and negative-tone) and HSQ resists, which are spin-cast onto commercially available TEM windows (approximately 50 µm x 50 µm) with SiN_x_ or SiO_2_ membranes with thicknesses ranging from 5 nm to 50 nm. One or more TEM windows are fabricated in a 3-mm diameter silicon handling frame (100 µm thick). Throughout this manuscript, we refer to the whole unit as the TEM chip and to the electron-beam transparent membrane as the TEM window.

1. Remove any organic residue from the TEM chip by performing O_2_ plasma cleaning for 30 s at 100 W (chamber pressure of 230 mT at approximately 5 sccm O_2_ flow).
2. Cleave a piece of silicon wafer, approximately 2 cm x 2 cm in size, to use as a holder for the TEM chip during resist spinning.
3. Place two stripes of double-sided carbon tape approximately equidistant from the center of the silicon holder and separated slightly less than the diameter of the TEM chip (see Figure 1). Rinse the stripes with isopropyl alcohol (IPA) to reduce their adhesive strength. This is necessary to avoid breaking the delicate TEM chip during removal from the Si holder.
4. Mount the TEM chip on the silicon holder making sure that it is attached to the carbon tape stripes only at two opposite edges as shown in Figure 1.

**Figure:**
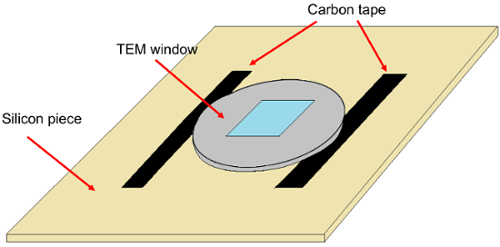

Figure 1**: TEM chip holder for resist spinning.** Notice that the TEM chip is attached to the silicon holder only at two edges to diminish the surface area contact, and hence, the adhesion force. Please click here to view a larger version of this figure.

### 2. Spin Coat Parameters for PMMA (Positive and Negative Tone) and HSQ Resists

Note: Resist thickness is not measured directly on the TEM chip, since it is small and usually the resist is cast on other thin layers (*e.g.,* Si film on SiO_2_ membrane), which complicates the measurement. Instead, resist thickness is determined by the spin speed calibrated using reflectometry measurements from films cast on a bulk Si sample. Reflectometry results were corroborated, usually with a precision better than 20%, by STEM top-down images of collapsed structures.

1. Mount the silicon holder on the spinner chuck and align the center of the TEM window approximately with the center of the spinner rotor.
2. Using a pipette, cover the entire TEM window with one drop (approximately 0.05 mL) of PMMA (A2 950K PMMA diluted in anisole to 0.5-1.0%) or HSQ (1% solids XR-1541).
3. Depending on the resist used, follow the spin coating and baking parameters shown in **Table 1**.
4. Carefully remove the TEM chip from the silicon holder. Inspect the resist uniformity over the TEM window using an optical microscope. If the film is homogenous across the central region of the membrane, proceed to the next step; otherwise, repeat the resist coating process on a fresh TEM window.

**Table:** 

**Resist**	**Spin speed (x g)**	**Film Thickness (nm)**	**Baking temperature (°C)**	**Baking time (min)**
Positive-tone PMMA	60	30	200^a^	2^a^
Negative-tone PMMA	60	15	200^a^	2^a^
HSQ	107	10	Not needed^b^	Not needed^b^
^a^see Ref.12; ^b^see Ref. 13

**Table 1: Resist spin coating and baking parameters.** Spin speed units in x g consider a 3-mm diameter TEM chip. Baking is performed on a hot plate for PMMA. No baking is needed for HSQ^13^. HSQ resist is stored refrigerated, so it needs to warm up to room temperature before spinning.

### 3. Load Sample in STEM, Map Window Coordinates, and Perform High-Resolution Focusing

1. Mount the resist-coated TEM chip on the STEM sample holder, making sure that the resist-vacuum interface faces the incoming beam, since the beam is optimally focused at the top of the sample. Also, make sure that the sides of the TEM window are aligned approximately with the x- and y-axis of the STEM stage. This will facilitate navigating to the TEM window.
  1. Load the TEM chip into the microscope, and pump overnight to reduce contaminants in the sample chamber.
2. Move the stage (x, y) coordinates such that the beam is more than 100 µm away from the center of the TEM window (to avoid accidental exposure). Set the STEM probe beam current and energy to 34 pA and 200 keV, respectively.
3. In diffraction mode imaging (stationary beam, z-contrast mode and mid-angle annular dark-field detector), set magnification to 30 kX with the beam out of focus, which makes it easier to find an edge of the TEM window. NOTE: The TEM window edges can also be found in imaging mode. We use diffraction mode because it is faster, since the beam does not need to be scanned to form an image.
4. Navigate towards the TEM window until an edge of the window is observed on the diffraction image. Navigate along the window edges and record the (x, y) coordinates of the four corners of the TEM window.
5. At the last window corner, increase magnification to 50 kX and perform rough focusing on the window membrane by moving the stage z-coordinate (z-height adjustment) until the crossover of the diffraction pattern orientation is observed. Subsequently, perform fine focusing by adjusting the objective lens current.
6. Increase magnification to 180 kX. Adjust focus, stigmation and aberration correction settings in order to obtain an aberration-corrected diffraction image of the window membrane as shown in Figure 2B. This focusing method is known as the Ronchigram method^14^.

**Figure:**
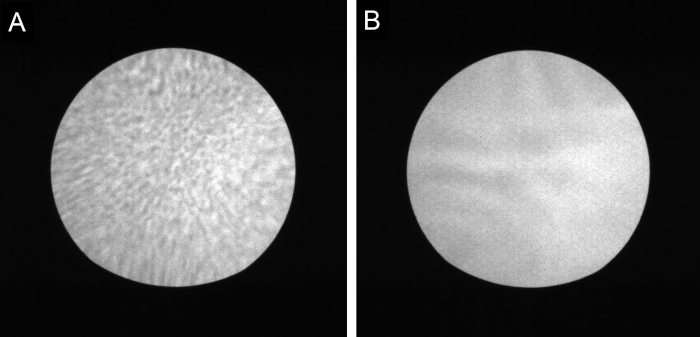

Figure 2**: Diffraction image of TEM window membrane.(A)** Focused but stigmatic image. The aberration-correction settings for this image are not optimum as evidenced by the closely-spaced diffraction fringes. **(B)** Exposure-ready non-stigmated image showing a smooth plateau diffraction pattern. Please click here to view a larger version of this figure.

### 4. Expose Patterns Using an Aberration-Corrected STEM Equipped with a Pattern Generator System.

Note: The aberration-corrected STEM used in this work is equipped with a pattern generator system (PGS), which controls the electron-beam position to expose patterns defined using computer aided design (CAD) software. Dose is controlled by defining the spacing between exposure points (step size) and the exposure time per point. **Table 2** summarizes the exposure parameters used in this protocol. Patterns are exposed at the center of the TEM window in "continuous mode," since the STEM used in this work does not include a beam blanker. Before and after the exposure, PGS positions the beam at any user-defined point in the field of view (FOV), preferably away from the pattern area. We use in this protocol the top right and bottom right corners of the FOV as the initial and final beam positions, respectively.

**Table:** 

**Resist**	**Dot exposure**	**Line exposure**	**Area exposure**
**Dose (fC/dot)**	**Step size (nm)**	**Dose (nC/cm)**	**Step size (nm)**	**Dose (µC/cm^2^)**
Positive tone PMMA	10-100	0.5	2–8	0.5	2,000
Negative tone PMMA	50-500	0.5	20–40	0.5	50,000–80,000
HSQ	10-100	0.5	10–20	0.5	20,000–30,000

**Table 2: Exposure parameters for PMMA (positive and negative tone) and HSQ resists.** The values shown are generic, since optimal dose values depend on the specific pattern design and targeted feature dimensions.

1. Close the beam gate valve to avoid any accidental exposure of the resist when moving the stage. Verify that the beam current is 34 pA and magnification is 180 kX.
2. Use the pre-recorded window corner coordinates to move the stage, so that the FOV center is 5 µm away from the center of the window. Open the beam gate valve and focus at this point using the Ronchigram method described in Step 3.6.
3. Close the beam gate valve. Move the stage to place the FOV at the center of the TEM window. Change magnification to 18 kX (corresponding to a 5 µm x 5 µm patterning FOV). Transfer the beam control to PGS and position the beam anywhere away from the pattern area (we use the top right corner in this protocol).
4. Perform the following actions in quick succession to avoid overexposing the resist at the initial and final beam positions.
  1. Open the gate valve and verify, by observing the beam diffraction pattern image, whether the beam is in focus at the initial beam position (as in Figure 2B). Expose the pattern.
  2. When the exposure is complete, check if the diffraction pattern image remains in focus at the final beam position. Finally, close the gate valve.
5. Remove the TEM chip from the STEM.

### 5. Resist Development and Critical Point Drying

Note: The development process depends on the resist used. Steps 5.1, 5.2, and 5.3 describe the developing process for positive-tone PMMA, negative-tone PMMA, and HSQ, respectively. However, all resists share the same final critical point drying process, which is necessary to avoid pattern collapse due to the high-aspect ratio of the patterns fabricated with this protocol. Critical point drying (CPD) uses liquid CO_2_ as working fluid, which is not miscible with water. Consequently, sample dehydration (steps 5.4-5.7) require the use of ACS reagent grade isopropyl alcohol (IPA).

1. Developing of positive-tone PMMA^15^: Prepare a 100-mL beaker with 3:1 solution of IPA:methyl isobutyl-ketone (MIBK). Place the beaker in a bath circulator at 0 °C (an ice bath at 0 °C is a lower cost alternative) and wait until the temperature is equalized. Grab the TEM chip with a pair of tweezers and gently stir it in the cold solution for 30 s. Proceed with Step 5.4.
2. Developing of negative-tone PMMA^16^: Gently stir the TEM chip in MIBK at room temperature (24 °C) for 2 min. Transfer the sample to an acetone solution and stir for 3 min. Proceed with step 5.4.
3. Developing of HSQ^13^: Stir the TEM chip in a "salty" deionized water solution, containing 1 wt% NaOH and 4 wt% NaCl, for 4 min at 24 °C. Stir the chip in pure deionized water for 2 min (to rinse off the salty developer). Proceed with Step 5.4.
4. Dip the TEM chip in ACS reagent grade IPA and gently stir it for 30 s.
5. Quickly place the TEM chip on the special 2" Si wafer shown in Figure 3A. Make sure that the TEM chip is always wet with IPA during the transfer. After approximately 2-3 min, close the CPD wafer holder assembly as depicted in Figure 3B. Leave the whole unit soaking in ACS reagent grade IPA for additional 15 min totally immersed in IPA.
6. Quickly transfer the complete CPD wafer holder assembly to a second container with fresh ACS reagent grade IPA and leave it for 15 min totally immersed in IPA.
7. Transfer the CPD wafer holder assembly to the CPD instrument process chamber (at all times the TEM chip should be totally immersed in IPA). Run the CPD process following the instrument's operating instructions.

**Figure:**
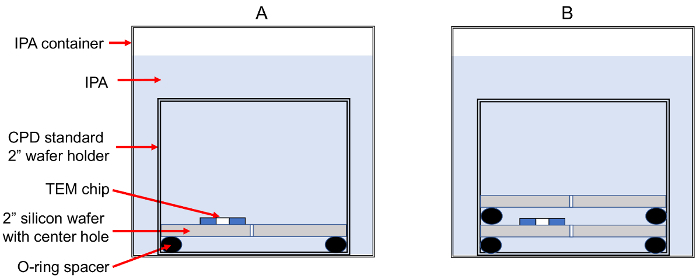

Figure 3**: In-house solution for the dehydration of TEM chips in a CPD standard 2" wafer holder. (A)** Schematic side view of the TEM chip on a special 2" Si wafer with a small hole drilled in the center (approximately 500 μm in diameter) to allow liquid flow. The wafer fits in a CPD standard 2" wafer holder supplied by the CPD system manufacturer. **(B)** A second special Si wafer encloses the TEM chip, thus reducing turbulent flow during the CPD process. In A and B, the CPD wafer holder is totally immersed in ACS reagent grade IPA. Please click here to view a larger version of this figure.

## Representative Results

Figure 4 shows lithographic patterns on positive-tone PMMA (resist removed from exposed regions after development) and negative-tone PMMA (resist removed from unexposed areas). TEM windows consisted of approximately 30 nm thick PMMA resist for positive-tone PMMA (15 nm thick for negative-tone PMMA) spin cast on a 5 nm thick SiN_x_ membrane. A thin metallic film (10 nm AuPd over 5 nm Ti) was deposited after development of positive-tone PMMA to enhance contrast during STEM imaging. For positive-tone PMMA, the average smallest isolated feature is 2.5 ± 0.7 nm (**Figure 4C, 4D**), while the smallest pitch pattern is 17.5 nm (Figure 4F). For negative-tone PMMA, the average smallest isolated feature is 1.7 ± 0.5 nm (Figure 4G), while the smallest pitch pattern is 10.7 nm (Figure 4J). 

**Figure:**
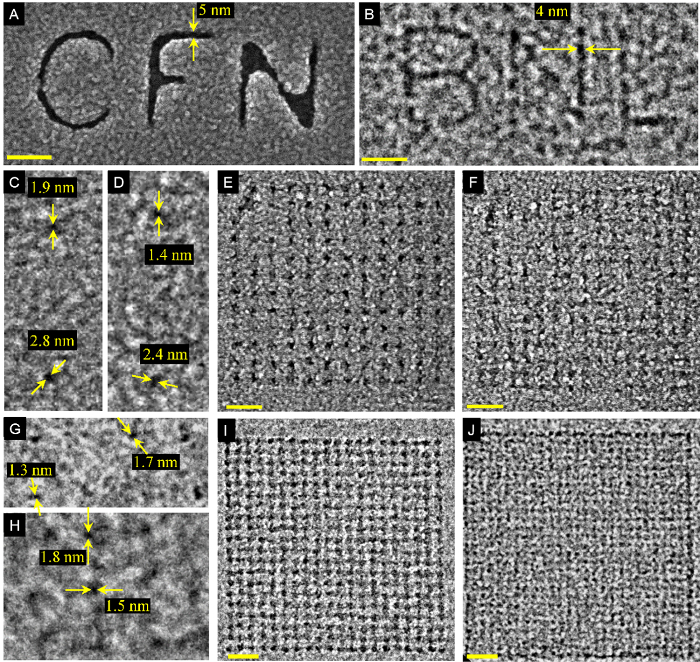

Figure 4**: Aberration-corrected electron beam lithography of positive- and negative-tone PMMA.** (A thin film of 10 nm AuPd over 5 nm Ti was deposited on all positive-tone PMMA patterns shown in this figure.) **(A)** SEM image of arbitrary patterns in positive-tone PMMA. **(B)** TEM image of arbitrary patterns in negative-tone PMMA. **(C,D)** SEM images of the smallest patterned holes in positive-tone PMMA, with average hole diameter of 2.5 ± 0.7 nm. **(E,F) **SEM images of hole arrays defined on positive-tone PMMA with a pitch of 21.5 nm (E) and 17.5 nm (F). **(G,H)** TEM images of pillar arrays in negative-tone PMMA with 20 nm pitch and with average pillar diameter of 1.7 ± 0.5 nm (G) and 1.8 ± 0.5 nm (H). **(I,J)** TEM images of negative-tone PMMA pillar arrays of 15.2 and 10.7 nm pitch, respectively. All scale bars are 40 nm. This figure has been reproduced from Manfrinato, V.R., Stein, A., Zhang, L., Nam, C.-Y., Yager, K.G., Stach, E.A, and Black, C.T. Aberration-Corrected Electron Beam Lithography at the One Nanometer Length Scale. *Nano Lett. ***17** (8), 4562-4567 (2017). Please click here to view a larger version of this figure.

Figure 5 displays patterns defined on HSQ resist. The TEM window used for HSQ lithography consisted of approximately 10 nm thick HSQ resist spin cast on a 27 nm thick Si membrane. After exposure and resist development, 3-4 nm of the ultra-thin Si layer in the HSQ-free regions (unexposed areas) of the window were removed by inductive coupled plasma (ICP) etching using a mixture of 50 sccm HBr and 20 sccm Cl_2_ gases at a chamber pressure of 10 mT (bias and ICP power of 60 W and 250 W, respectively). Figure 5A consists of four rows of short vertical lines. The upper two rows were exposed with a line dose stepped exponentially from 2 to 120 nC/cm (0 nm designed width for these lines). The lower two rows were exposed with an area dose stepped exponentially from 3,000 to 60,000 μC/cm^2^ (5 nm wide and 200 nm long designed rectangles). Figure 5B is a zoomed image of the center region of the bottom row in Figure 5A. The two leftmost, the four center, and the four rightmost lines were exposed with an area dose of 23,300, 27,300, and 32,000 μC/cm^2^, respectively. The four center lines have an average measured width of 7 nm.

**Figure:**
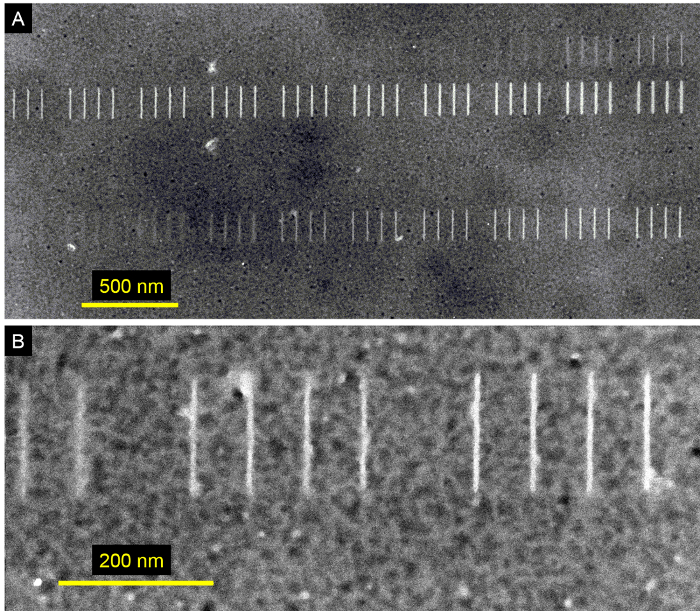

Figure 5**: Aberration-corrected electron beam lithography of HSQ resist.** (The TEM window used in this figure was made of 27 nm-thick Si. After HSQ development, inductive coupled plasma etching was used to remove 3-4 nm of Si from the areas not covered by HSQ.) **(A)** TEM image of four rows of vertical lines exposed with doses varying exponentially from 2 to 120 nC/cm (top two rows) and 3,000 to 60,000 μC/cm^2^ (bottom two rows). The beam step size was 0.5 nm for all lines. **(B)** High magnification TEM image of the central area of the bottom row in (A). The group of 4 lines in the center have an average measured width of 7 nm and were exposed with an area dose of 27,300 μC/cm^2^. Please click here to view a larger version of this figure.

## Discussion

The most critical step in the protocol is focusing the electron beam before exposure. This is necessary to achieve highest-resolution patterning. When performing multiple exposures (*e.g.,* when a TEM chip has multiple windows and each is being patterned), it is important to refocus the beam before each exposure at a distance of at-most 5 μm from the exposure area. The protocol also includes steps to check the beam focus before and after exposure at two extreme positions of the patterning area (top and bottom corners), which allows a determination of whether some defocusing occurred during patterning, for example due to a membrane being locally tilted in the patterning region.

Another important step in this protocol is using critical point drying (CPD) to dry samples after developing the exposed resist patterns. Without this step, patterns will frequently collapse due to the high aspect ratio of the patterned structures (*i.e.,* patterned resist lateral dimensions smaller than the thickness). Most CPD systems supply a standard 2" wafer holder. However, since TEM chips are very small and the patterned structures are quite delicate, they might be damaged during the CPD process when placed in holders designed for larger samples. Figure 3 shows an in-house solution for CPD of TEM chips using a standard wafer holder. The two wafers, with a flow-enabling hole at the center, enclose the TEM chip and protect it from turbulent flow during the CPD process.

The determination of the optimal resist film thickness tries to balance competing requirements. On the one hand, it should be as thin as possible to achieve the highest resolution and to avoid pattern collapse, but on the other hand, it should be thick enough for pattern transfer applications such as lift-off and etching. This protocol uses 1% HSQ, which is the lowest dilution commercially available and whose further dilution in the lab is not recommended (our experience shows that diluted HSQ often leads to partial crosslinking). However, since diluted PMMA does give reproducible results, this protocol uses 1% for positive-tone PMMA (30 nm thickness) and 0.5% and 1% for negative tone (15 and 30 nm thickness, respectively). We have found that positive-tone PMMA resist does not suffer from pattern collapse as negative-tone PMMA does, thus the use of thinner thickness for negative tone as shown in **Table 1.** In addition, negative-tone PMMA has ~50% thickness loss after e-beam exposure (and before development), so the final thickness for negative-tone PMMA is ~7 to 15 nm. (The 1.7 and 1.8 nm features from Figure 4 have about 7 nm resist thickness, which is at the limit of pattern collapse.) The PMMA patterns shown in Figure 4 did not use a CPD step; however, if available, this protocol recommends the use of CPD after development of PMMA patterns. In contrast, we found CPD to be critical for HSQ processing due to the fact that it cannot be further diluted (to achieve thinner thickness) and because thicker HSQ patterns are needed to use as an etching mask (*e.g.,* to etch silicon as shown in Figure 5).

The positive-tone PMMA patterns in Figure 4 were coated with a thin metallic film to increase contrast during imaging. The Supporting Information in the work of Manfrinato* et al*.^1^ shows that the effect of this metallic coating on the metrology of the patterns is negligible. Similarly, we consider that the results shown in Figure 5 for HSQ resist do not depend drastically on the particular choice of TEM window structure based on the ultra-thin thickness of the underlying Si layer.

To the best of our knowledge, all the measurements described in the Representative Results Section for positive- and negative-tone PMMA^1^ (Figure 4) are the smallest features reported in the literature to date^1^,^7^,^12^,^16^,^17^. Manfrinato *et al*.^1^ also demonstrated sub-5 nm pattern transfer, from the resist to a target material, using conventional metal lift-off (for positive-tone PMMA) and sequential infiltration synthesis^18^ of ZnO (for negative-tone PMMA). The results shown in Figure 5 for HSQ are not the smallest reported features^7^. However, this protocol is useful for obtaining reproducible features in HSQ at resolutions better than 10 nm, and demonstrates single-digit patterning of silicon structures.

The protocol presented here describes a process for patterning arbitrary structures with single digit nanometer resolution using the conventional electron-beam resists PMMA and HSQ. Additionally, the results shown here and in Ref. 1 demonstrate that such patterns can be transferred with high fidelity to a target material of choice.

## Disclosures

The authors have nothing to disclose.
